# Clinical Outcome of Conversion Surgery for Stage IV Esophageal Cancer Following Chemoradiation

**DOI:** 10.3390/biomedicines13030745

**Published:** 2025-03-18

**Authors:** Hu-Lin Christina Wang, Ke-Cheng Chen, Pei-Ming Huang, Chih-Hung Hsu, Chia-Hsien Cheng, Feng-Ming Hsu, Ta-Chen Huang, Jhe-Cyuan Guo, Jang-Ming Lee

**Affiliations:** 1 Division of Trauma, Department of Surgery, Far Eastern Memorial Hospital, New Taipei City 220216, Taiwan; drcwang@gmail.com; 2 College of Computer Science and Engineering, Yuan Ze University, Taoyuan 320315, Taiwan; 3 Division of Thoracic Surgery, Department of Surgery, National Taiwan University Hospital and National Taiwan University College of Medicine, Taipei 100233, Taiwan; cskchen@gmail.com (K.-C.C.); e370089@gmail.com (P.-M.H.); 4 Department of Oncology, National Taiwan University Hospital, Taipei 100225, Taiwan; chihhunghsu@ntu.edu.tw (C.-H.H.); jasoncheng@ntu.edu.tw (C.-H.C.); hsufengming@gmail.com (F.-M.H.); e360215@gmail.com (T.-C.H.); jhecyuanguo@gmail.com (J.-C.G.)

**Keywords:** esophageal cancer, metastatic, surgery

## Abstract

**Purpose:** We aimed to identify the impact of conversion surgery to survival in patients with stage IV esophageal cancer who have a stabilized disease and good treatment response before surgery. **Patients and Methods:** This retrospective study included patients with esophageal cancer M1 disease treated at a tertiary medical center from April 2002 to June 2021. For patients with a good clinical response to chemoradiation and well-controlled metastatic lesions, esophagectomy and lymphadenectomy were performed. A propensity score-matching (PSM) study with a 1:2 ratio and based on patient age, tumor stage, and metastasis status was conducted for verifying the results. **Results:** We enrolled 162 patients, including 124 treated with concurrent chemoradiation therapy (CCRT) alone and 38 treated with CCRT followed by esophagectomy. A total of 114 patients were analyzed using PSM, including 76 patients treated with CCRT alone and 38 patients treated with CCRT and surgery. The 3- and 5-year OS was 24.6% vs. 2.8% and 12.3% vs. 1.4% (*p* = 0.006), and PSM was 24.6% vs. 4.6% and 12.3% vs. 2.3% (*p* = 0.033) for those with or without esophagectomy, respectively. Multivariate analysis revealed surgery with esophagectomy as an independent prognostic factor for OS with odd ratios (95% confidence interval [CI]) of 1.91 (1.23–2.95) (*p* = 0.004). **Conclusions:** Surgical resection following CCRT holds a potential survival benefit for the patients with a favorable response to CCRT for patients with stage IV esophageal cancer.

## 1. Introduction

Esophageal cancer is a common cancer and a serious malignancy owing to its poor prognosis and mortality rate. Despite newer tools of diagnosis and treatment modalities, esophageal cancer is still characterized by a high degree of locoregional and distant recurrence, with poor overall survival and approximately 40% of the patients presenting with metastatic disease (M1) at the time of first diagnosis [1,2]. Systemic chemotherapy has been the standard treatment for these patients who have esophageal squamous cell carcinoma (ESCC) with distant organ metastasis. When combined with radiation therapy, this has been shown to improve survival, with or without surgery [3]. However, survival outcomes are often poor, with a median survival of less than 1 year [4,5,6]. There has been substantial improvement in survival outcomes in the multidisciplinary treatment of unresectable ESCC [7,8]. The addition of immunotherapy agents such as pembrolizumab and nivolumab plus chemotherapy have shown improved progression-free survival, higher response rates and overall survival rates over 1 year in patients with advanced esophageal cancer [7,8]. For local control of metastatic lesions, various ablation techniques such as radiofrequency ablation and stereotactic body radiation therapy (SBRT) can be used to provide a quick but endurable outcome for tumor eradication [9,10]. These progresses in treatment may stabilize the disease status for a prolonged period of time and spare room for conversion therapy with radical surgical resection. It is therefore according to the updates of the ESSO core curriculum that multimodality treatment including systemic and local abrasion therapy might improve the survival of esophageal cancer with oligo-metastasis [11].

Conversion surgery, which involves performing surgical resection after a favorable response to neoadjuvant chemotherapy, has shown potential survival benefits for patients with stage IV gastric or gastroesophageal junction cancer which is originally considered as unresectable disease status. Studies including the AIO-FLOT3 trial and retrospective analyses suggested that selected patients can achieve improved survival outcomes with this approach, highlighting its potential as an effective treatment strategy [12,13,14].

The rationale of conversion surgery was deemed possible due to a few key factors, as mentioned above. Moreover, the advent of minimally invasive esophagectomy has been demonstrated to be feasible for complete tumor eradication with a significant reduction in surgical morbidity [15,16]. For the patients of esophageal cancer with nodal metastasis in the M1 area without distant organ metastasis, surgical resection can significantly improve the survival following a nodal downstaging with neoadjuvant therapy [17,18]. Thus, integrating treatment modalities to include conversion surgery may therefore be justified to enhance control of both primary and metastatic disease for stage Iv esophageal cancer [12,18,19].

For such patients who have shown improved tumor responses to multimodal treatment, we hypothesize that conversion surgery may have a role in improving the survival of these patients. For this study, we aim to analyze the survival outcomes of conversion surgery for patients who initially presented with metastatic disease and treated with definitive chemoradiation therapy with those who only received definitive chemoradiation therapy.

## 2. Patients and Methods

This study enrolled a cohort of patients diagnosed with stage IV esophageal cancer who had tumor metastasis to distant organs, including lymph node metastasis detected in the retroperitoneum, which could not be eradicated by surgery, from April 2002 to June 2021 in a tertiary medical center. The work was conducted and reported in line with the STROCSS criteria [20]. Clinical data included personal demographics, associated risk factors, tumor pathology and location, neoadjuvant and adjuvant chemotherapy and/or radiation therapy, date of disease recurrence, and TNM staging [9,10,21]. Regular clinical follow-up was performed for all patients who underwent imaging and endoscopic examination every 3 to 6 months after treatment. Disease recurrence or progression was defined as locoregional disease, distant disease, or both according to the imaging and endoscopic studies. The number of metastatic organs was determined. The Research Ethics Committee of National Taiwan University Hospital, Taipei, Taiwan, approved this retrospective study (202211084RIND) by 13 February 2023 and waived the requirement for informed consent.

### 2.1. Treatment

All patients were treated with definitive cisplatin plus 5-fluoro-uracil or cisplatin plus taxane-based concurrent chemoradiation therapy (CCRT). Radiotherapy was delivered via either IMRT (intensity modulated radiation therapy) or VMAT (volume modulated arc therapy) to the primary esophageal tumor, lymph nodes, and metastatic organs. Additional ablation therapy, including radiofrequency ablation, microwave ablation, or surgical resection, was administered for the metastatic lesion(s) after discussion in the multidisciplinary meeting.

All of the patients received a series of staging studies before and after treatment including whole body computed tomography (CT), position emission tomography (PET), and endoscopic ultrasound (EUS). For the tumor at the middle and upper third esophagus, bronchoscopy was performed to exclude tracheal invasion by the tumor. Magnetic resonance imaging (MRI) or CT-guided biopsy were optionally performed for confirmation of the metastatic lesion before or after treatment. The protocols and duration of chemotherapy after CCRT with or without surgery was decided according to the tumor response to treatment and the presence of residual disease after treatment.

Esophagectomy and esophageal reconstruction were performed once a favorable response to treatment had been achieved according to the following criteria: (1) resectability of the primary tumor after CCRT, (2) acceptable risk of surgery according to the general performance of the patients, and (3) feasibility of controlling metastatic lesions by local ablation therapy including complete surgical resection, radiofrequency or microwave ablasion or chemoradiation, which was confirmed by subsequent imaging studies.

Open or minimally invasive McKeown (cervical anastomosis) or Ivor Lewis (intrathoracic anastomosis) esophagectomy with three-field lymphadenectomy were performed for the surgical treatment of the patients according to the availability of a proximal resection margin during surgery [15,16].

### 2.2. Statistics and Data Analysis

Statistical analyses were performed using SPSS version 28 (IBM, Armonk, NY, USA) and XLSTAT v.2022.3.1.1377 (Addinsoft, Paris, France). The descriptive statistical characteristics of patients with and without surgical resection were summarized and compared. Chi-squared or Fisher’s exact test was used to compare categorical variables. Kaplan–Meier survival analysis was also performed for the overall survival (OS) and progression-free survival (PFS) of the two groups of patients. PFS was defined as the time from treatment initiation to the confirmation of disease progression. The OS was defined as the time from treatment initiation to patient death. Cox proportional hazards regression analysis was used to calculate hazard ratios with 95% confidence intervals. Propensity score matching was performed to minimize the effects of selection bias (caliper width 0.1 of the standard deviation of the propensity score). Only patients with squamous cell carcinoma or adenocarcinoma were included in the propensity score-matching (PSM) analysis. The patients were matched for age, sex, cell type, smoking, alcohol consumption, betel nut risk factors, metastatic status, and clinical staging. Patients who underwent surgery after CCRT were matched to those who received CCRT using a 1:2 greedy approach [22]. The Chi-square test was used to compare patients and tumor characteristics in both matched and unmatched cohorts.

## 3. Results

**Figure 1:**
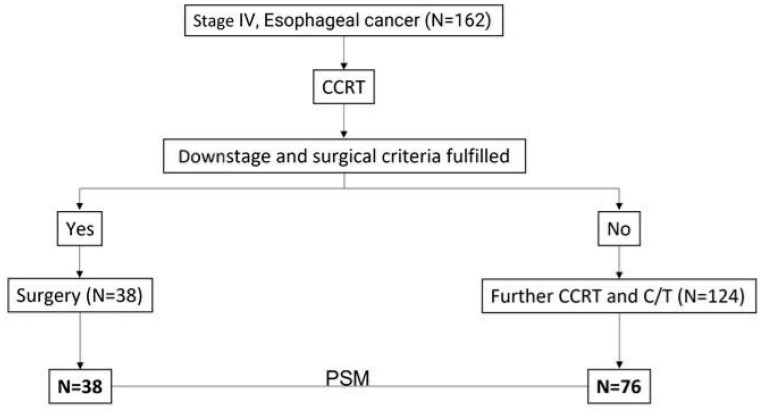
The process of patient selection in the study. PSM: propensity score-matching study.

**Figure 2:**
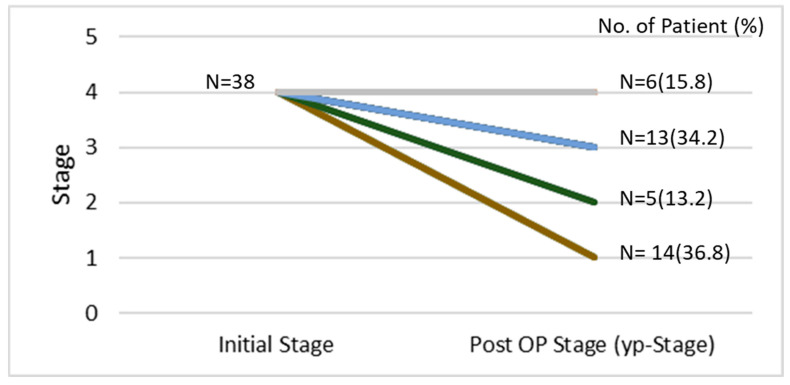
The initial and new staging (yp-stage) after CCRT and esophagectomy in the surgical group.

**Figure 3:**
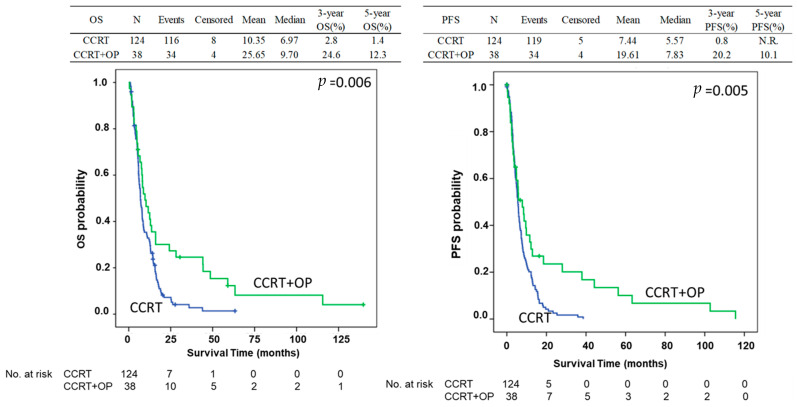
Overall survival (OS) and progression-free survival (PFS) curves of patients with or without esophagectomy in the entire study cohort of stage IV esophageal cancer with distant metastasis. The patients who underwent esophagectomy had significantly better OS and PFS than those who did not (*p* = 0.006 and 0.005, respectively). CCRT: concurrent chemoradiation therapy; OP: operation.

**Figure 4:**
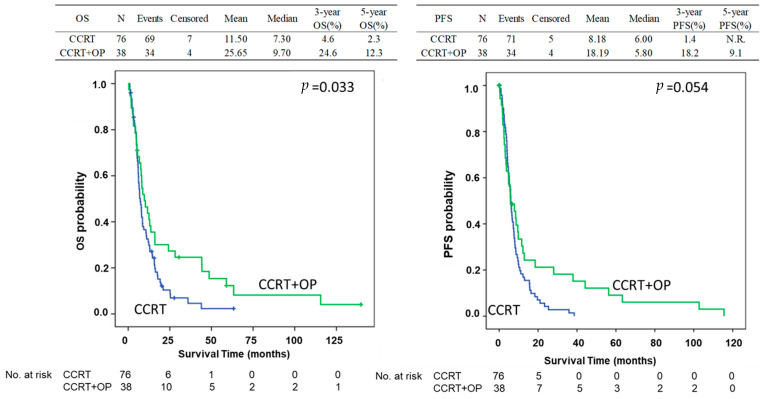
Overall survival (OS) and progression-free survival (PFS) curves of patients with or without esophagectomy after propensity score-matched study cohort of stage IV esophageal cancer with distant metastasis. The patients who underwent esophagectomy had significantly better OS and borderline improved PFS than those who did not (*p* = 0.033 and 0.054, respectively). CCRT: concurrent chemoradiation therapy; OP: operation.

**Figure 5:**
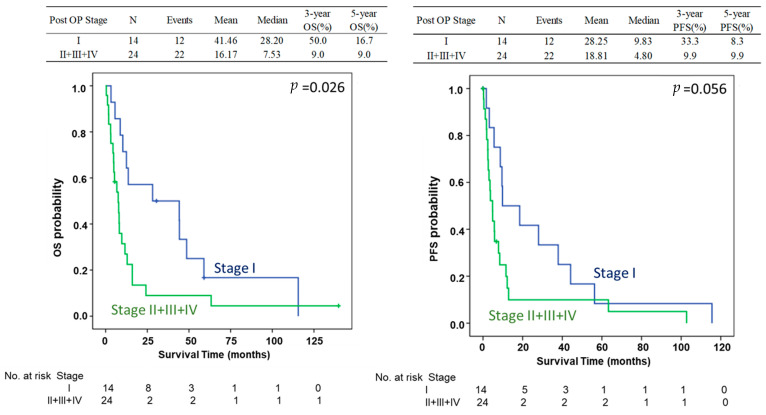
Overall survival (OS) and progression-free survival (PFS) curves of patients in the surgical group according to the yp-staging of the tumor. For the patients with yp-stage I, the OS and PFS is significantly or marginally better than those who with other yp-stages (II, III, and IV) (*p* = 0.033 and 0.054, respectively).

In total, 162 patients with esophageal cancer stage IV and M1 disease were enrolled in this study with 124 and 38 patients treated with CCRT alone and CCRT followed by esophagectomy, respectively. Among these patients, 114 were selected in the PMS analysis including 76 and 38 patients with CCRT alone and CCRT followed by esophagectomy, respectively. The process of patient selection is summarized in the flowchart of Figure 1. The clinical demographics of all patients are shown in Table 1. Most patients were under 65 years of age (range: 26–91 years); 92% of the patients were males. There were no differences in age or sex between the CCRT only and CCRT plus surgery groups. Most of the patients had the cell type of squamous cell carcinoma (*n* = 134, 88.7%) and 15 patients had adenocarcinoma (9.9%). Most of the patients (66.7%) had an initial clinical stage of cT3, and 89% had positive N staging. The clinical profiles of the patients including recurrences according to the location, number and organs of metastatic organs, and distribution of co-mobility are shown in Table 1. Most of the patients have downstaging of the tumor after CCRT. For the patients receiving esophagectomy, there were 7.9% (*n* = 3), 21.1% (*n* = 8), and 63.2% (*n* = 24) with three, two, and one organs with distant metastasis before treatment, respectively, including 7.9% (*n* = 3), 68.4% (*n* = 26), 15.8% (*n* = 6), and 34.2% (*n* = 13) patients with metastasis in the lung, abdomen, bone, and neck, respectively. The initial and new staging after CCRT and esophagectomy (yp-stage) was demonstrated in Figure 2 with 36.8%, 13.2%, 34.2%, and 15.8% for yp-staging of I, II, III, and IV, respectively (Figure 2). The mean overall survival was 10.35 months in the CCRT group vs. 25.65 months in the CCRT plus surgery group in the whole study of patients (*p* < 0.05). The mean progression-free survival was 7.44 vs. 17.83 months (*p* < 0.05). The 3-year and 5-year overall survival (OS) was 24.6% and 12.3% for CCRT-plus-surgical patients, and 2.8% and 1.4% for CCRT patients (*p* = 0.006). The 3-year and 5-year progression-free survival (PFS) was 20.2% and 10.1% for CCRT-plus-surgical patients, and 0.8% and 0.0% for CCRT patients (*p* = 0.005) (Figure 3). Univariate analysis (Table 2) identified CCRT without surgery as a significant factor associated with significantly worse OS and PFS (OR: 1.77; 95% confidence interval: 1.17–2.66 for CCRT plus surgery vs. CCRT alone *p* = 0.006 of OS; OR: 1.82; 95% confidence interval: 1.19–2.77, *p* = 0.006 for CCRT plus surgery vs. CCRT alone of PFS) (*p* < 0.05). The difference was observed both in the overall (OS) and progression-free survival duration (PFS) (*p* < 0.05) (Table 3). A similar finding was observed in the multivariable analysis, with significantly worsening OS and PFS of the patients with CCRT alone (OR: 1.91; 95% confidence interval: 1.23–2.95 *p* = 0.004 for OS; OR: 1.74; 95% confidence interval: 1.12–2.70, *p* = 0.014 for PFS) (Table 3). A total of 114 patients were included in the propensity score-matching analysis, with 76 patients in the CCRT group and 38 in the CCRT plus surgery group. There were no differences in patient demographics between the two groups (Table 4). In the propensity score-matching cohort, the mean overall survival was 11.5 months in the CCRT group vs. 25.65 months in the CCRT plus surgery group (*p* = 0.033). The mean progression-free survival was 8.18 vs. 18.19 months (*p* = 0.054). The 3-year and 5-year overall survival (OS) was 24.6% and 12.3% for CCRT-plus-surgical patients, and 4.6% and 2.3% for CCRT only patients. The 3-year and 5-year progression-free survival (PFS) was 18.2% and 9.1% for CCRT-plus-surgical patients, and 1.4% and 0.0% for CCRT only patients (Figure 4). Figure 5 reveals the OS and PFS curves of the surgical group according to the yp-staging of the tumor. For the patients with yp-stage I, the OS is significantly better than those with other yp-stages (II, III, and IV), whereas a similar trend was observed for PFS without statistical significance (with a median OS and PFS of 41.46 m vs. 16.17 m and 28.25 m vs. 18.81 m, *p* = 0.026 and 0.056, respectively). There were 26 and 12 patients with distant lymph node metastasis only or organ metastasis in the surgical group, respectively, and there was no statistical difference in overall survival between the patients with nodal or organ metastasis (*p* = 0.43, Supplementary Figure S1). There was also no statistical difference in overall survival between the patients enrolled in the early and late cohort of the current study (*p* = 0.785) (Supplementary Figure S2).

The perioperative complications were shown in Table 5 with overall, anastomotic leakage, and pulmonary complication rates of 36.8% (*n* = 14), 5.3% (*n* = 2), and 13.2% (*n* = 5), respectively. There was no surgical mortality in these patients after surgery.

## 4. Discussion

This retrospective study using single-center in-hospital data from 2001 to 2021 revealed significant overall survival and progression-free survival for selected patients with metastatic esophageal cancer treated with CCRT and conversion surgery. The findings were consistent with the previous study, which was previously shown to have potential survival benefits for patients with stage IV gastric or gastroesophageal junction cancer [12,13,14]. This difference was observed both for the OS and PFS in the multivariable analysis and propensity score-matching studies. Although there is no surgical mortality in the current study, for these patients receiving surgery after high dose thoracic radiation, pulmonary complications (13.2%, *n* = 5) remained the most frequent problems encountered after surgery. We have routine use of cortical steroid for three days after surgery and prolonged or restarted this treatment if suspicious pulmonary lesions detected in chest imaging and infection excluded after clinical studies, similar to the strategies for acute respiratory distress syndrome [23].

Igaue et al. reported the clinical outcome of surgery for the patients with lymph node metastasis classified in the M1 regions and demonstrated an equivalent survival outcome between the M0 and M1 groups once a complete lymphadenectomy was achieved [17], suggesting the potential survival benefit of conversion surgery for certain patients classified in the M1 status. Sugimura et al. report a multi-institutional study in Japan of surgical outcome for patients of stage IV esophageal cancer including 51 (77%) patients with nodal metastasis and 15 (23%) with distal organ metastasis, and show an excellent survival outcome with 3- and 5-year overall survival rates of 32.4% and 24.4%, respectively [18]. In 2017, a retrospective review of 52 patients with metastatic esophageal adenocarcinoma showed that chemoradiation prior to surgical resection showed a 5-year survival of 6% [24]. In our study of stage IV esophageal cancer with distant organ metastasis, the survival outcome stands between that observed in the western countries and Japan [17,24]. The 5-year overall survival rate of the surgical group after CCRT was 12% and progression-free survival of 5 years was 8.9%. A significant difference was detected in the overall survival rate between the surgical plus CCRT and CCRT alone groups both in the whole and propensity-score matching cohorts. The survival difference in the surgical group is even more prominent in the patients with yp-stage I disease with a significantly better OS as compared to those who with other yp-stages. The survival benefit of esophagectomy appears to correlate with the response to neoadjuvant treatment in patients with metastatic esophageal cancer.

The main reason for the survival difference between the two groups of patients might be attributed to the aggressive local ablation therapy for both of the primary and metastatic lesions including surgery. According to the national cancer database of the United State of America, aggressive radiation with a definitive dose improved overall survival for the patients with metastatic esophageal cancer as compared to those who underwent palliative dose radiation [25]. In an Asian study, it was demonstrated that 36.6% of the patient with esophageal cancer was detected to have airway involvement and 13.1% of whom developed tracheoesophageal fistulae (TEF), which associated with a poorer survival outcome [26]. A better primary tumor control by complete esophagectomy can therefore reduce the risk of mortality attributed to the local tumor invasion. In addition, an effective local abrasion both for the primary and metastatic sites can reduce the tumor burden of the disease which can be correlated and monitored with circulating tumor DNA and hence reduce the risk of disease progression. Another possible reason could be that most patients underwent minimally invasive esophagectomy (MIE) using combined thoracoscopic and laparoscopic approaches. There were 36.8% of the patients with surgical complication including two patients with anastomotic leakage (5.1%) and five with pneumonia (12.8%), but there was no surgical mortality in the current surgical group. As compared to surgery for patients with operable disease in initial diagnosis of the Western population-based surgical results which reveal anastomotic leakage and a pulmonary complication rate ranging from 10 to 20% and 30 to 45%, respectively [27], conversion surgery following definitive chemoradiation seems not to increase surgical morbidity and mortality. A smooth postoperative recovery can make the patient more responsive to subsequent treatment, enhancing their survival without detriment from the surgical intervention.

This study was limited by the small sample size of surgically treated patients which might make the PFS unable to reach a significant difference in the PMS cohort, although a significant survival impact on PFS can be observed in the model of multiple logistic regression for the whole study population. For further confirmation of these findings, an international multi-institutional study is ongoing for data collection and analysis now. Other limitations of this study include selection bias in patients undergoing surgery with evaluation of the surgical risk before surgery which is inherent to retrospective studies and the functional characteristics of the patients including the ECOG performance status was not available in the patients enrolled in the patient cohort. We tried to minimize the effect by the propensity score-matching study. However, there were several confounders including tumor response to CCRT, and following the method of adjuvant therapy, our observations should be confirmed in a larger prospective randomized trial. As the study enrolled the patients before the era of immunotherapy, the role of surgery would therefore be more prominent with the improved treatment response and overall survival of the patients with stage IV esophageal cancer with an introduction of immunotherapy into systemic therapy for treating these patients [7,8]. Likewise, the evolution of various abrasion therapies including radiation, radio-frequency, or microwave abrasion can also enhance the efficacy of eradication of the metastatic lesion and facilitate the performance of conversion surgery with time. However, the study cohort from 2001 to 2021, in which period the extent of esophagectomy and protocol of chemoradiation remained similar in the hospital and therefore all of the patients fulfilling the criteria were included in the study in that similar surgical and non-surgical strategies were adopted in the caring of the patients. In particular, the overall survival of the patients in the surgical group was found to be similar between the patients enrolled in the early and late cohorts of the study.

## 5. Conclusions

Surgical resection following CCRT holds a potential survival benefit for the patients with favorable response to CCRT for patients with stage IV esophageal cancer especially for the patients with yp-stage I disease after esophagectomy. Our findings suggest the need for further large-scale studies to investigate the role and indication of conversion surgery for patients with stage IV esophageal cancer included in the multiple treatment modalities.

## Figures and Tables

**Table 1:** Clinical characteristics of the whole study participants with stage IV esophageal cancer with distant metastasis.

Variable	Total	CCRT Only	CCRT + OP	*p*-Value
	*n* = 162	*n* = 124	*n* = 38	
**Age**				0.058
<65	112 (69.1)	81 (65.3)	31 (81.6)	
≥65	50 (30.9)	43 (34.7)	7 (18.4)	
**Gender**				1
Female	13 (8.0)	10 (8.1)	3 (7.9)	
Male	149 (92.0)	114 (91.9)	35 (92.1)	
**Cell type**				0.739
SCC	134 (88.7)	101 (89.4)	33 (86.8)	
Adenocarcinoma	15 (9.9)	10 (8.8)	5 (13.2)	
Small cell	1 (0.7)	1 (0.9)	0	
Others	1 (0.7)	1 (0.9)	0	
**Chemotherapy**				0.028
5Fu + cisplatin	4 (19.0)	4 (30.8)	0	
Taxol or Taxotere + cisplatin	8 (38.1)	3 (23.1)	5 (62.5)	
5Fu + Taxol or Taxotere + cisplatin	7 (33.3)	6 (46.2)	1 (12.5)	
other	2 (9.5)	0	2 (25.0)	
**cT**				0.132
cT1	3 (1.9)	2 (1.6)	1 (2.6)	
cT2	6 (3.7)	5 (4.0)	1 (2.6)	
cT3	108 (66.7)	77 (62.1)	31 (81.6)	
cT4	33 (20.4)	30 (24.2)	3 (7.9)	
Unkown	12 (7.4)	10 (8.1)	2 (5.3)	
**cN**				0.729
Negative	6 (3.7)	4 (3.2)	2 (5.3)	
Positive	144 (88.9)	110 (88.7)	34 (89.5)	
Unkown	12 (7.4)	10 (8.1)	2 (5.3)	
**Tumor site**				0.523
Upper	19 (16.4)	13 (16.5)	6 (16.2)	
Middle	30 (25.9)	23 (29.1)	7 (18.9)	
Lower	42 (36.2)	27 (34.2)	15 (40.5)	
Multiple	24 (20.7)	16 (20.3)	8 (21.6)	
Other *	1 (0.9)	0	1 (2.7)	
**Number of metastatic organs**				0.205
0 **	23 (14.2)	20 (16.1)	3 (7.9)	
1 organ	78 (48.1)	54 (43.5)	24 (63.2)	
2 organs	47 (29.0)	39 (31.5)	8 (21.1)	
3 or more organs	14 (8.6)	11 (8.9)	3 (7.9)	
**Co mobility ^&^**				0.556
No	100 (61.7)	75 (60.5)	25 (65.8)	
Yes	62 (38.3)	49 (39.5)	13 (34.2)	

* Esophagogastric junction; ** Retroperitoneal para aortic lymph node metastasis; CCRT: concurrent chemoradiation therapy; OP: operation; SCC: squamous cell carcinoma; 5Fu: 5-fluoro-uracil; ^&^: DM, hypertension, COPD, renal failure, and CVA, etc.

**Table 2:** Univariate analysis for the significant factors of overall survival of stage IV esophageal cancer with distant metastasis.

Variable	Total	Overall Survival	*p*-Value	Progression-Free Survival	*p*-Value
	*n* = 162	HR (95% CI)		HR (95% CI)	
**Age**					
<65	112	1		1	
≥65	50	0.82 (0.57–1.16)	0.260	1.00 (0.70–1.41)	0.977
**Gender**					
Female	13	1		1	
Male	149	1.18 (0.62–2.15)	0.645	1.31 (0.70–2.43)	0.399
**Cell type**					
SCC	134	1		1	
Adenocarcinoma	15	0.73 (0.41–1.29)	0.276	0.61 (0.25–1.11)	0.105
Small cell	1	2.37 (0.33–17.16)	0.393	1.82 (0.25–13.12)	0.554
Others	1	0.81 (0.11–5.81)	0.834	0.58 (0.08–4.13)	0.583
Chemotherapy **					
5FU + cisplatin	4	1		1	
Taxol or Taxotere + cisplatin	8	0.30 (0.09–1.07)	0.064	0.39 (0.11–1.37)	0.141
5FU + Taxol or Taxotere + cisplatin	7	0.36 (0.10–1.34)	0.128	0.38 (0.10–1.39)	0.143
other	2	0.28 (0.05–1.67)	0.163	0.46 (0.08–2.63)	0.382
**cT**					
cT1	3	1		1	
cT2	6	1.73 (0.41–7.29)	0.456	2.50 (0.59–10.58)	0.213
cT3	108	2.12 (0.67–6.71)	0.203	2.55 (0.80–8.10)	0.113
cT4	33	2.29 (0.69–7.55)	0.174	3.42 (1.03–11.31)	0.044
Unknown	12	2.47 (0.68–8.91)	0.168	4.22 (1.17–15.23)	0.028
**cN**					
Negative	6	1		1	
Positive	144	1.01 (0.44–2.29)	0.988	1.10 (0.48–2.51)	0.817
Unknown	12	1.19 (0.44–3.24)	0.731	1.75 (0.66–4.68)	0.262
**Tumor site**					
Upper	19	1		1	
Middle	30	1.39 (0.77–2.49)	0.275	1.16 (0.65–2.08)	0.619
Lower	42	0.68 (0.38–1.20)	0.185	0.53 (0.30–0.92)	0.025
Multiple	24	1.54 (0.83–2.84)	0.172	1.21 (0.65–2.23)	0.548
Other *	1	1.77 (0.23–13.42)	0.579	0	0.968
**Recurrence**					
0	22	1		1	
Locoregional	31	0.79 (0.44–1.43)	0.433	0.82 (0.45–1.48)	0.512
distant	73	0.86 (0.52–1.42)	0.550	0.90 (0.54–1.50)	0.695
Local and distant metastasis	36	0.76 (0.43–1.34)	0.336	1.01 (0.58–1.77)	0.974
**Number of metastatic organs**					
0 **	23	1		1	
1 organ	78	0.87 (0.52–1.45)	0.582	0.81 (0.49–1.35)	0.417
2 organs	47	0.95 (0.55–1.62)	0.841	0.97 (0.56–1.65)	0.898
3 or more organs	14	0.91 (0.45–1.84)	0.791	1.47 (0.74–2.94)	0.275
**OP**					
CCRT + OP	38	1		1	
No (only CCRT)	124	1.77 (1.17–2.66)	0.006	1.82 (1.19–2.77)	0.006

* Esophagogastric junction; ** Retroperitoneal para aortic lymph node metastasis; CI: confidence interval; CCRT: concurrent chemoradiation therapy; HR: hazard ratio; OP: operation; SCC: squamous cell carcinoma; 5FU: 5-fluoro-uracil.

**Table 3:** Multivariate analysis for the significant factors of overall survival of stage IV esophageal cancer with distant metastasis.

Variable	Total	Overall Survival	*p*-Value	Progression-Free Survival	*p*-Value
	*n* = 162	HR (95% CI)		HR (95% CI)	
**Age**					
<65	112	1		1	
≥65	50	0.75 (0.51–1.09)	0.127	0.96 (0.66–1.40)	0.841
**Gender**					
Female	13	1		1	
Male	149	1.07 (0.55–2.06)	0.850	1.24 (0.64–2.39)	0.520
**cT**					
cT1	3	1		1	
cT2	6	2.06 (0.48–8.83)	0.332	2.74 (0.64–11.70)	0.173
cT3	108	2.33 (0.70–7.71)	0.166	2.82 (0.86–9.31)	0.088
cT4	33	2.19 (0.64–7.55)	0.213	3.31 (0.97–11.32)	0.057
Unkown	12	1.65 (0.40–6.76)	0.487	3.45 (0.85–14.09)	0.084
**cN**					
Negative	6	1		1	
Positive	144	0.77 (0.32–1.84)	0.551	0.83 (0.35–1.98)	0.671
**OP**					
CCRT + OP	38	1		1	
No (only CCRT)	124	1.91 (1.23–2.95)	0.004	1.74 (1.12–2.70)	0.014

CI: confidence interval; CCRT: concurrent chemoradiation therapy; HR: hazard ratio; OP: operation.

**Table 4:** Clinical characteristic of patients in CCRT only and CCRT plus surgery group after propensity-score matching analysis.

Variable	Total	CCRT Only	CCRT + OP	*p*-Value
	*n* = 114 (%)	*n* = 76 (%)	*n* = 38 (%)	
**Age**				0.176
<65	84 (73.7)	53 (69.7)	31 (81.6)	
≥65	30 (26.3)	23 (30.3)	7 (18.4)	
**Gender**				0.684
Female	7 (6.1)	4 (5.3)	3 (7.9)	
Male	107 (93.9)	72 (94.7)	35 (92.1)	
**Cell type**				0.757
SCC	101 (88.6)	68 (89.5)	33 (86.8)	
Adenocarcinoma	13 (11.4)	8 (10.5)	5 (13.2)	
**cT**				0.142
cT1	3 (2.6)	2 (2.6)	1 (2.6)	
cT2	5 (4.4)	4 (5.3)	1 (2.6)	
cT3	76 (66.7)	45 (59.2)	31 (81.6)	
cT4	22 (19.3)	19 (25.0)	3 (7.9)	
Unknown	8 (7.0)	6 (7.9)	2 (5.3)	
**cN**				0.775
Negative	4 (3.5)	2 (2.6)	2 (5.3)	
Positive	102 (89.5)	68 (89.5)	34 (89.5)	
Unknown	8 (7.0)	6 (7.9)	2 (5.3)	
**Tumor site**				0.654
Upper	15 (16.9)	9 (17.3)	6 (16.2)	
Middle	22 (24.7)	15 (28.8)	7 (18.9)	
Lower	32 (36.0)	17 (32.7)	15 (40.5)	
Multiple	19 (21.3)	11 (21.2)	8 (21.6)	
Other *	1 (1.1)	0	1 (2.7)	
**Number of metastatic organs**				0.248
0 **	12 (10.5)	9 (11.8)	3 (7.9)	
1 organ	58 (50.9)	34 (44.7)	24 (63.2)	
2 organs	36 (31.6)	28 (36.8)	8 (21.1)	
3 or more organs	8 (7.0)	5 (6.6)	3 (7.9)	

* Esophagogastric junction; ** Retroperitoneal para aortic lymph node metastasis; CCRT: concurrent chemoradiation therapy; OP: operation; SCC: squamous cell carcinoma.

**Table 5:** Peri-operative Complications.

Perioperative Complication of the Patient After Esophagectomy	Total *n* = 38 (%)
**Complication (Overall)**	14 (36.8)
Leakage	2 (5.3)
Hoarseness	3 (7.9)
Pulmonary	5 (13.2)
Others *	4 (10.5)

* Four have the symptoms as follow: Shortness of breath (1 (2.6)), Acute renal failure (1 (2.6)), Left neck wound swelling (1 (2.6)), Hiatal hernia (1 (2.6)), Left main bronchus laceration s/p primary repair (1 (2.6)), Abdominal distention (1 (2.6)).

## Data Availability

The original contributions presented in this study are included in the article/Supplementary Material. Further inquiries can be directed to the corresponding author.

## Supplementary Materials

The following supporting information can be downloaded at https://www.mdpi.com/article/10.3390/biomedicines13030745/s1. Figure S1: Overall survival (OS) curves of patients of the surgical group according to the metastatic status by lymph node metastasis only or organ metastasis. No significant difference was detected between the two groups of the patients (*p* = 0.43); Figure S2: Overall survival (OS) curves of patients of the surgical group according to enrollment period (before and after 2013). No significant difference was detected between the two groups of the patients (*p* = 0.785).
