# Supplementary material for: Clinical Outcome of Conversion Surgery for Stage IV Esophageal Cancer Following Chemoradiation
Source: Biomedicines. 2025 Mar 18;13(3):745. doi: 10.3390/biomedicines13030745 (PMC11940810; doi:10.3390/biomedicines13030745)
Supplement: Supplementary file 1 [file biomedicines-13-00745-s001.zip › biomedicines-3486107-supplementary.pdf]

| Metastatic | N | Events | Mean | Median | 3-year OS(%) | 5-year OS(%) |
|---|---|---|---|---|---|---|
| LN meta | 26 | 24 | 26.80 | 12.50 | 26.9 | 9.0 |
| Organ meta | 12 | 10 | 23.75 | 6.73 | 19.4 | 19.4 |

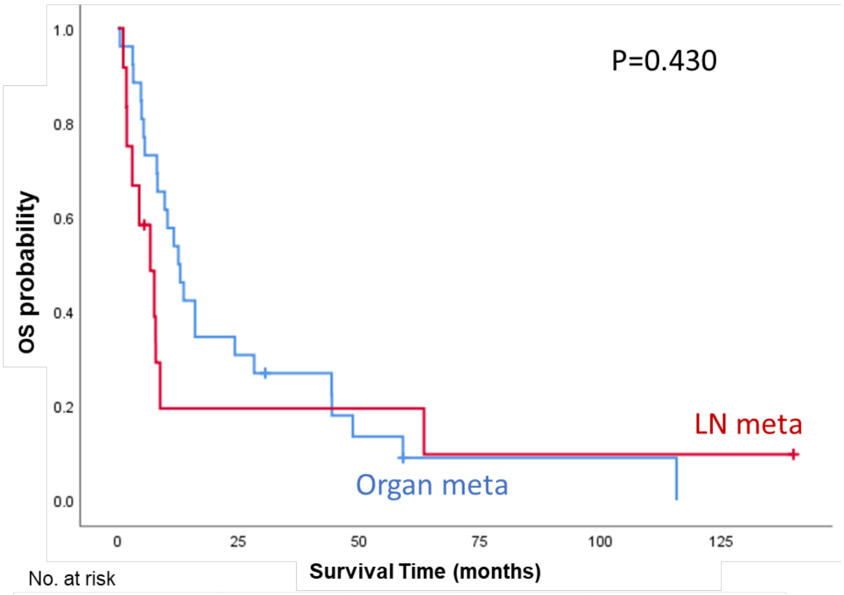


No. at risk

| | | | | | | |
|---|---|---|---|---|---|---|
| Lymgh | 26 | 8 | 3 | 1 | 1 | 0 |
| Others | 12 | 2 | 2 | 1 | 1 | 1 |

Supplementary Figure S1

| OS | N | Events | Mean | Median | 3-year OS(%) | 5-year OS(%) |
|---|---|---|---|---|---|---|
| Before 2013 | 81 | 79 | 15.40 | 7.30 | 10.6 | 4.0 |
| After 2013 | 81 | 71 | 12.18 | 7.87 | 6.0 | 6.0 |

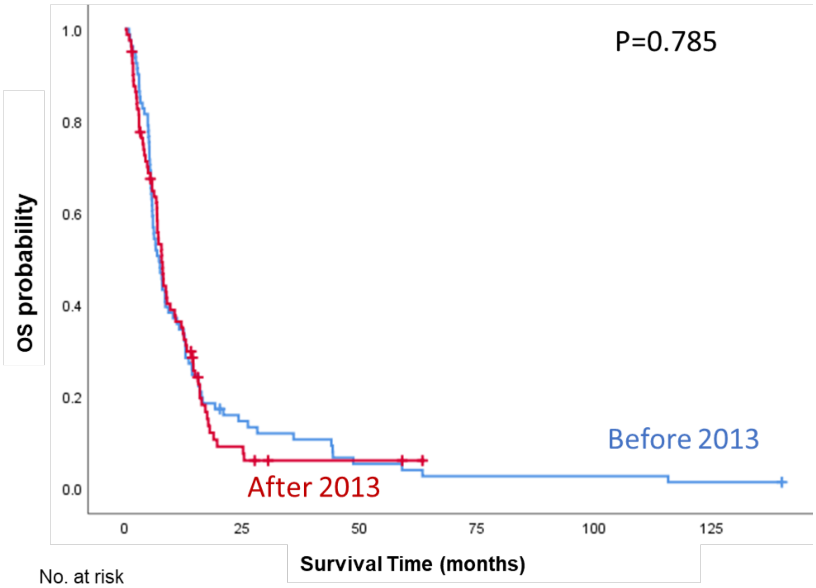


No. at risk

| | | | | | | |
|---|---|---|---|---|---|---|
| Before 2013 | 81 | 11 | 4 | 2 | 2 | 1 |
| After 2013 | 81 | 6 | 2 | 0 | 0 | 0 |

Supplementary Figure S2
